# Caspase-dependent signaling underlies glioblastoma cell death in response to the fungal metabolite, fusarochromanone

**DOI:** 10.3892/ijmm.2014.1842

**Published:** 2014-07-09

**Authors:** ELAHE MAHDAVIAN, MONIQUE MARSHALL, PATRICK M. MARTIN, PATRICE CAGLE, BRIAN A. SALVATORE, QUINCY A. QUICK

**Affiliations:** 1 Department of Chemistry and Physics, LSU-Shreveport, Shreveport, LA 71115, USA; 2 Department of Biology, North Carolina A&T State University, Greensboro, NC 27411, USA; 3 Department of Biological Sciences, Tennessee State University, Nashville, TN 37209, USA

**Keywords:** fusarochromanone, poly(ADP-ribose) polymerase, glioblastoma

## Abstract

Fungal metabolites continue to show promise as a viable class of anticancer agents. In the present study, we investigated the efficacy of the fungal metabolite, fusarochromanone (FC101), for its antitumor activities in glioblastomas, which have a median survival of less than two years and a poor clinical response to surgical resection, radiation therapy and chemotherapy. Using clinically applicable doses, we demonstrated that FC101 induced glioblastoma apoptotic cell death via caspase dependent signaling, as indicated by the cleavage of poly(ADP-ribose) polymerase, glioblastoma (PARP). FC101 also induced differential reactive oxygen species (ROS) levels in glioblastoma cells, contrasting a defined role of oxidative stress in apoptotic cell death observed with other fungal metabolites. Furthermore, the antitumorigenic effects of FC101 on tumor cell migration were assessed. Cell migration assays revealed that FC101 significantly reduced the migratory capacity of glioblastomas, which are incredibly invasive tumors. Taken together, the present study establishes FC101 as a candidate anticancer agent for the cooperative treatment of glioblastomas.

## Introduction

Fungal metabolites are naturally occurring compounds produced by fungi with known anticancer properties (1). This is supported by early studies on cytochalasins, fungal metabolites that have been shown to inhibit the growth and migration of prostate cancer cells (2), as a consequence of impairing the biophysical function of actin filaments by binding and capping their barbed ends, thus, preventing microfilament elongation. Additionally, secondary metabolites (phenolic compounds, melanins and lanostane-type triterpenoids) produced by the white rot fungus, *Inonotus obliquus*, have also exhibited antitumorigenic properties in breast, leukemia and gastric cancers (3–5). Collectively, these studies provide experimental evidence for the persistent exploration and utility of fungal metabolites as agents for the treatment of human cancers.

In the present study, we evaluated the efficacy of fusarochromanone (FC101), a fungal metabolite produced by *Fusarium equiseti* (6), in glioblastoma, the most common and aggressive type of malignant primary brain tumor. To date, few studies have been performed that have assessed the efficacy of FC101 as an anticancer agent. However, experimental studies conducted by Furmanski *et al* (7) and Dréau *et al* (8) have demonstrated that FC101 inhibits the growth of breast cancer cells and the *in vitro* and *in vivo* growth of melanomas. In addition a recent study by our group [Mahdavian *et al* (unpublished data)] also demonstrated that FC101 exerts antitumor affects in prostate and bladder cancer, further establishing the anticancer activity of this fungal metabolite. To this end, although the usage of fungal metabolites as potential therapeutic agents for the treatment of glioblastomas has received little attention, in this study, we provide evidence of the antitumor effects of FC101 on glioblastoma cell proliferation through the promotion of apoptotic cell death, as well as its antagonizing affects on glioblastoma cell migration.

## Materials and methods

### Cell conditions and reagents

U87, A172 and U251 glioblastoma cells were purchased from the American Type Culture Collection (ATCC; Manassas, VA, USA). All cell lines were maintained in Dulbecco’s modified Eagle’s medium-DMEM (Invitrogen, Grand Island, NY, USA) containing 10% fetal bovine serum (FBS; Invitrogen), 2 mM L-glutamine (Invitrogen), 100 nM MEM non-essential amino acids (Invitrogen) and penicillin-streptomycin (Invitrogen) at 37°C and 5% CO_2_. FC101 was generously provided by Dr Elahe Mahdavian (Department of Chemistry and Physics, LSU-Shreveport, Shreveport, LA, USA).

### Crystal violet cell proliferation assay

The cells were plated in 24-well plates, treated with 10, 5, 2.5 and 1 μM FC101 and allowed to incubate for 48 h (vehicle controls were treated with PBS) for dose-response experiments. For time-course experiments, the cells were treated with 1 μM FC101 and allowed to incubate for 24, 48, 72 and 96 h. Subsequently, the tissue culture medium was removed, the cell monolayer was fixed with 100% methanol for 5 min and stained with 0.5% crystal violet in 25% methanol for 10 min. The cells were then washed 3 times for 5 min each with distilled water to remove excess dye and allowed to dry overnight at room temperature. The incorporated dye was then solubilized in 0.1 M sodium citrate (Sigma-Aldrich, St. Louis, MO, USA) in 50% ethanol. Subsequently, 100 μl of treated and control samples were transferred to 96-well plates and optical densities were read at 540 nm using an xMark Microplate absorbance spectrophotometer (Bio-Rad Laboratories, Hercules, CA, USA).

### Cell motility

Motility assays were conducted according to the manufacturer’s instructions (Cell Biolabs Inc., San Diego, CA, USA). A cell suspension containing 0.5–1.0x10^6^ cells/ml was prepared in serum-free mediaum with the vehicle (PBS) or 1 μM FC101, while 500 μl of medium containing 10% fetal bovine serum was added to the lower chamber of the migration plate. Cell suspension (300 μl) containing the vehicle or 1 μM FC101 was then added to the inside of each insert and allowed to incubate for 24 h at 37°C and 5% CO_2_. Subsequently, the non-migratory cells were removed from the plate inserts (as per the manufacturer’s instructions), and the migratory cells were counterstained, solubilized and the optical density densities were read at 560 nm using an xMark Microplate absorbance spectrophotometer (Bio-Rad Laboratories).

### Measurement of reactive oxygen species (ROS; H_2_O_2_) generation

ROS assays were conducted in accordance with the manufacturer’s instructions (Cell Biolabs Inc.). A serial dilution of H_2_O_2_ (0–100 μM) was prepared to generate a standard curve, while the U87 and A172 cells (1x10^7^) were plated, exposed to 1 μM FC101 or the vehicle (PBS) for 24 h and sonicated. Standards and samples were then mixed with an aqueous working reagent (as per the manufacturer’s instructions), incubated on a shaker for 30 min at room temperature, and the optical densities were read at 595 nm with a xMark Microplate absorbance spectrophotometer (Bio-Rad Laboratories).

### ELISA

The cells were plated and treated with 1 μM FC101 for 24 h or the vehicle (PBS); the cells were then lysed with CelLytic M Cell lysis reagent (Sigma-Aldrich) and protein concentrations were determined using the Bradford method. Protein extracts were diluted to a final concentration of 20 μg/ml in PBS, coated to the wells of a PVC microtiter plate, and allowed to incubate at 4°C overnight. Wells containing protein antigen were then washed with PBS 3 times, blocked with 5% non-fat dry milk in PBS for 2 h, and washed again with PBS 2 times. Subsequently, incubation with α-actinin 1 (Abnova, Walnut, CA, USA) or α-actinin 4 (Abnova) antibodies was performed overnight at 4°C followed by washing 4 times with PBS. Incubation with an HRP-conjugated secondary antibody was performed for 3 h, followed by 4 washes with PBS. TMB substrate solution (Thermo Fisher Scientific, Inc., Rockford, IL, USA) was added for 15 min followed by a stop reaction with 0.2 M sulfuric acid. An absorbance reading at 450 nm was performed with a xMark Microplate absorbance spectrophotometer (Bio-Rad Laboratories). Absorbance readings were normalized to FC101 and the vehicle-treated samples that were processed with secondary antibody only.

### Western blot analysis

The cells were plated and treated with 1 μM FC101 for 24 h or the vehicle (PBS), rinsed with PBS, and lysed with CelLytic M Cell lysis reagent (Sigma-Aldrich). Protein concentrations were subsequently determined using the Bradford method. Proteins were separated by SDS-PAGE in 8% polyacrylamide gels, then transferred to PVDF membranes. For immunoblotting, the PVDF membranes were incubated with poly(ADP-ribose) polymerase (PARP) and caspase-9 antibodies (Cell Signaling Technology, Danvers, MA, USA) recognizing target proteins overnight at 4°C. The membranes were then incubated with an HRP-conjugated secondary antibody (1:2,000) for 1 h at room temperature, analyzed using the enhanced chemiluminescence (ECL) detection system (Thermo Fisher Scientific) and visualized by autoradiography. Tubulin (1:5,000) was used as a loading control.

## Results

### Antiproliferative effects of FC101 on glioblastomas

**Figure 1:**
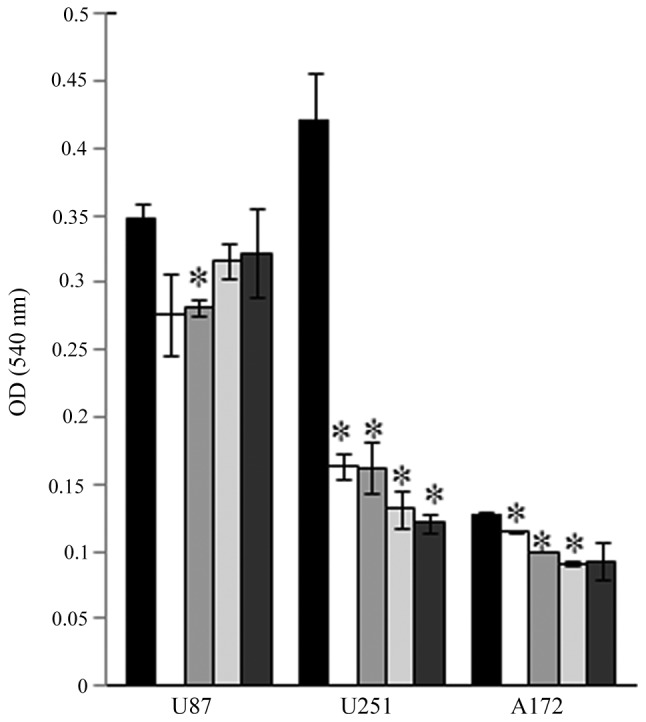
Fusarochromanone (FC101) inhibits glioblastoma cell proliferation. Vehicle-treated control cells (black bars); and cells treated with 10 μM (white bars); 5 μM (medium gray bars); 2.5 μM (light gray bars); 1 μM (dark gray bars). Data shown are representative of 3 independent experiments performed in duplicate (means ± SE) showing similar results (^*^P<0.05).

**Figure 2:**
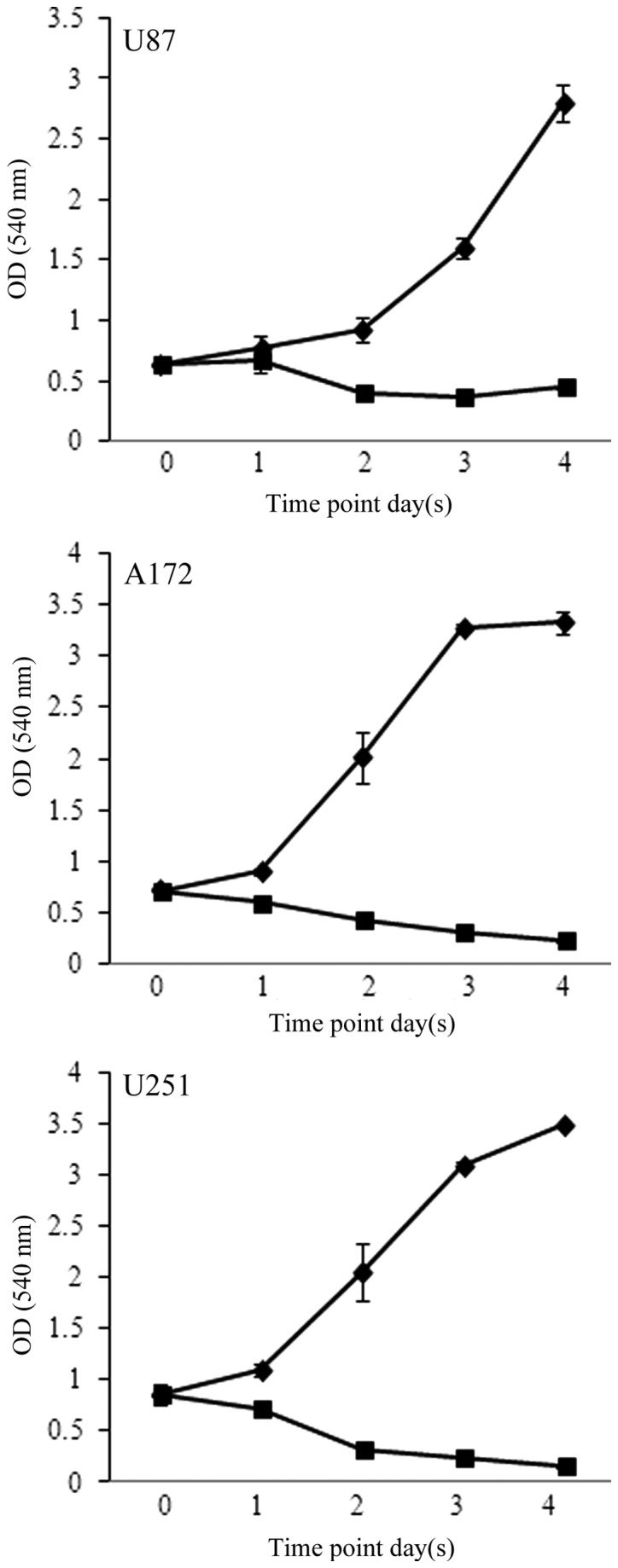
Time-course analysis of fusarochromanone (FC101)-treated glioblastoma cells. Glioblastoma cells underwent cytotoxity-associated cell death in response to 1 μM FC101 (square), as compared to the vehicle-treated control cells (diamond). Shown is an experiment representative of 3 independent experiments performed in duplicate (means ± SE) that displayed similar results.

Our initial experiments evaluated the effects of FC101 on glioblastoma cell proliferation in dose-response experiments that showed that FC101 (10, 5, 2.5 and 1 μM) decreased the proliferative capacity of the U87, A172 and U251 cells (Fig. 1). The most demonstrative effects of FC101 in dose-response experiments were observed in the U251 cells which displayed a statistically significant decrease (P<0.05) in cell proliferation, even when treated with the lowest dose of 1 μM FC101, as compared to the vehicle-treated control cells (Fig. 1). To further assess the effects of FC101 on glioblastoma cell proliferation, time-course experiments were performed in which the cells were treated with 1 μM FC101 and examined over a 4-day period (Fig. 2). Consistent with the results from the dose-response experiments, time-course analysis also revealed that FC101 reduced the proliferation of the glioblastoma cells, as evidenced by a pronounced decrease in cell proliferation 96 h post-FC101 exposure, as compared to the vehicle-treated control cells examined at the same time point (Fig. 2). Additionally, ANOVA analysis of the time-course data revealed that the statistically significant (P<0.05) decrease observed in glioblastoma cell proliferation following treatment with FC101 was likely a consequence of a cytotoxic cellular response. This was determined by comparing glioblastoma cell viability at day 0 and 4 days post-FC101 exposure, which showed a 31, 66 and 82% decrease in U87, A172 and U251 cell proliferation, respectively (Fig. 2). These data are in accordance with those of previous studies on FC101 in melanomas and breast cancer (7,8), as well as with those of studies conducted on glioblastomas with the fungal metabolites, ophiobolin A (9) and fumonisin B1 (10–12) that also induced cytotoxic cellular responses.

### FC101 induces glioblastoma cell death via apoptosis

**Figure 3:**
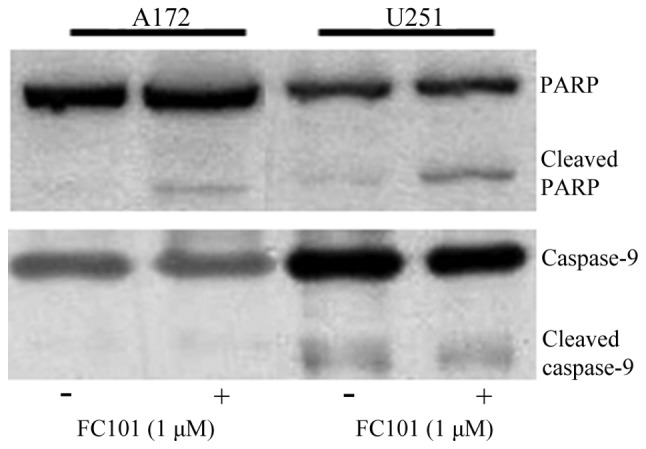
Fusarochromanone (FC101) induces apoptotic cell death. FC101 promoted programmed cell death as indicated by the expression of cleaved PARP in the A172 and U251 cells treated with 1 μM FC101 for 24 h. Immunoblots shown are representative of 3 independent experiments that showed similar results.

**Figure 4:**
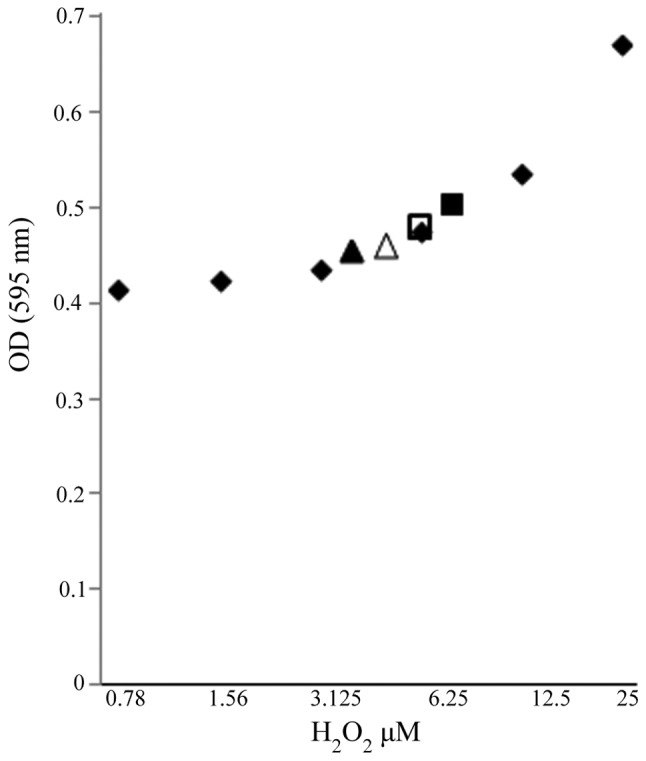
Detection of ROS in A172 (triangle) and U251 (square) cells treated with 1 μM fusarochromanone (FC101) for 24 h as determined by measuring H_2_O_2_ concentration. H_2_O_2_ standard serial dilution (diamond); vehicle-treated control cells (solid symbols); cells treated with 1 μM FC101 (open symbols). Data displayed is an average of 3 separate independent experiments.

To determine the mechanisms underlying the cytotoxic cellular response described above, we assessed the involvement of programmed cell death-associated signaling proteins and oxidative stress-related molecules in response to FC101. PARP, an enzymatic protein that plays a role in DNA repair and a downstream target of caspases, which mediate programmed cell death, was examined as an indicator of apoptosis in glioblastoma cells treated with FC101. Immunoblotting procedures revealed that the glioblastoma cells exposed to 1 μM FC101 produced cleaved PARP protein expression, a marker of cells undergoing apoptosis (Fig. 3), while no changes in caspase-9 protein expression, an initiator caspase, were observed (Fig. 3). Additionally, ROS, known inducers of death receptor and mitochondrial-mediated apoptosis in cancer cells, were also examined as a mechanistic contributor to FC101-induced glioblastoma cell death. To this end, we observed a modest increase in the hydrogen peroxide concentration in the A172 cells treated with FC101 as compared to the vehicle-treated control cells, while the U251 cells displayed a decrease in hydrogen peroxide concentration following treatment with FC101 (Fig. 4). Taken together, these data demonstrate that FC101 induces a differential oxidative stress response that is cell type-dependent and that this fungal metabolite promotes apoptotic cell death in glioblastoma cells via caspase signaling.

### Inhibition of glioblastoma cell migration

**Figure 5:**
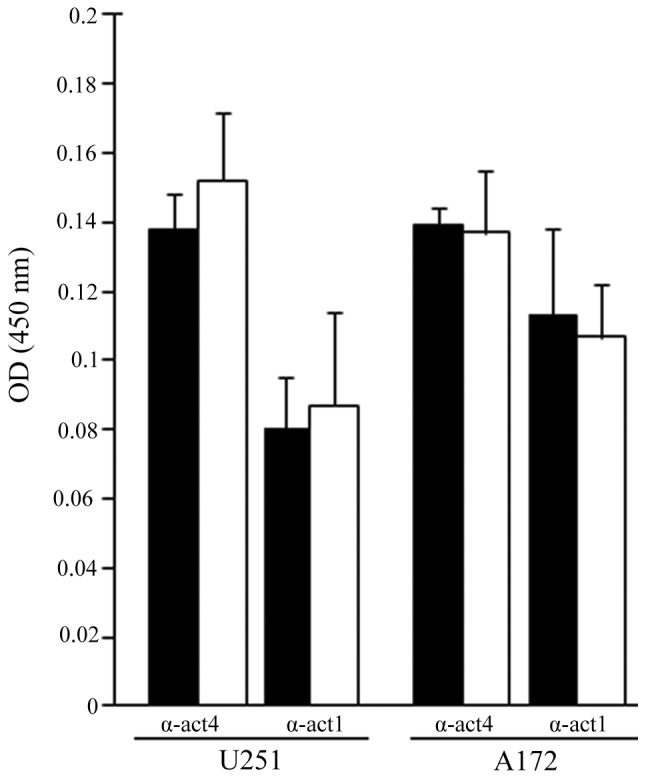
ELISA of α-actinin 1 and 4 (α-act1 and α-act4) in A172 and U251 cells 24 h post-exposure to 1 μM fusarochromanone (FC101). Data displayed are an average (means ± SE) of 3 separate independent experiments. Vehicle-treated control cells (black bars); cells treated with 1 μM FC101 for 24 h (white bars).

**Figure 6:**
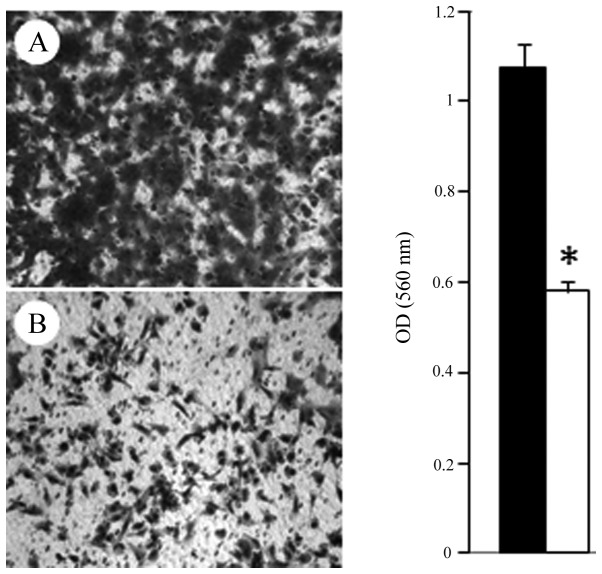
Fusarochromanone (FC101) impairs glioblastoma cell migration. Glioblastoma cell migration was significantly (^*^P<0.05) diminished in the U251 cells treated with 1 μM FC101 for 24 h. Data shown are representative of 3 independent experiments performed in duplicate (means ± SE) showing similar results.(A) ehicle-treated control cells (black bars); (B) cells treated with 1 μM FC101 for 24 h (white bars).

Glioblastomas are highly invasive tumors that ultimately result in high rates of disease recurrence. Therefore, in this study, we analyzed the ability of FC101 to affect glioblastoma cell migration and structural proteins involved in this cellular process. α-actinin 1 and 4, actin-binding proteins with an established role in cell migration (13,14) and shown to be expressed at high levels in glioblastomas as compared to normal brain tissue (14), were examined in the FC101-treated glioblastoma cells. Using immunoenzyme linked assays, a moderate increase in α-actinin 1 and 4 expression was observed in the U251 cells exposed to FC101 when compared to the vehicle-treated control cells, while a diminutive decrease in α-actinin 1 and 4 expression was observed in the A172 cells treated with FC101 (Fig. 5). Subsequently, cell migration assays revealed that 1 μM FC101 induced a 46% decrease (P<0.05) in glioblastoma cell migration as compared to the vehicle-treated control cells (Fig. 6). These results are in accordance with the findings of a recent study by Bury *et al* (15), that also showed that the fungal metabolite, fusicoccin A, impaired the migratory and invasion ability of glioblastoma cells.

## Discussion

Fungal metabolites are emerging as promising anticancer agents for the treatment of human cancers, in part, since they are naturally derived products. However, the utility of metabolic products from fungi as adjuvant or neoadjuvant agents for the treatment of brain tumors, specifically glioblastomas, has received little attention. To date, the most well characterized fungal metabolite exhibiting cytotoxic anticancer activity in glioblastomas is the fumonisin, fumonisin B1, which exerts its biological effects by interfering with sphingolipid metabolism (10–12). Additionally, studies on the fungal metabolites, brefeldin A and ophiobolin A, which impair the structure and functions of the Golgi apparatus and calmodulin, respectively, have also shown that they promote glioblastoma cell death (9,16). Collectively, these studies establish the cytotoxic antitumor effects of fungi-produced metabolites in glioblastomas by affecting a range of biological processes.

In this study, we demonstrated that the fungal metabolite, FC101, exhibited antitumorigenic affects in glioblastomas as a consequence of inducing cell death. This was supported by cell proliferation data from the present study that displayed an IC_90_ in glioblastomas treated with 1 μM FC101 and examined 4 days later. Our findings are in accordance with those from other studies on breast, melanoma and bladder cancers, that also showed that therapeutic applicable concentrations of FC101 were effective at promoting tumor cell killing in these human cancers (7,8). In this study, it was also determined that FC101 induced caspase-dependent apoptotic glioblastoma cell death, as indicated by PARP cleavage, a caspase target. This is consistent with data from other studies on melanomas and breast cancer, that also demonstrated caspase-dependent apoptosis as the underlying mechanism of tumor cell death observed in these cancers in response to FC101 [(8) and study by Mahdavian *et al* (unpublished data)]. Although PARP cleavage provided substantial evidence that FC101 promoted caspase-dependent apoptotic glioblastoma cell death, changes in caspase-9 protein expression were not detected. The lack of an affect on caspase-9 expression supports the notion that FC101 does not induce apoptotic cell death in glioblastomas via an intrinsic mechanism, as suggested in in another study of ours [Mahdavian *et al* (unpublished data)], which showed that FC101 had no effect on intrinsic apoptotic pathway proteins (BAD, BAK and BAX) in breast cancer cells. In addition to caspase signaling, oxidative stress has also been shown to act as a mechanistic contributor of fungal metabolite-induced apoptotic cell death (10,11,17,18). Stockmann-Juvala *et al* (10) and Chaudhari *et al* (17) demonstrated that fumonisin B1 and the trichothecene, T-2 toxin, elicited apoptotic cell death as a consequence of increased ROS production and reduced glutathione levels in cervical cancer and glioblastomas, respectively. However, the assessment of oxidative stress in our study revealed differential ROS levels in the A172 and U251 glioblastoma cells treated with FC101 as compared to the control-treated cells, suggesting that variations in the genetic backgrounds of these cells likely contribute to the divergent responses observed and the role of oxidative stress in FC101-induced apoptotic glioblastoma cell death.

In this study, we further demonstrated that FC101 also exhibits antitumor properties in glioblastomas by abrogating cell migration, a cell behavior that contributes to the metastatic invasiveness of human cancers, ultimately leading to disease recurrence. This observation is consistent with recent studies on the fungal metabolites, fusicoccin A and chaetoglobosin A that also had antimigratory effects on glioblastoma and leukemia cells which were attributed to the impairment of the actin cytoskeleton (15,19). However, in this study, the analysis here of α-actinin 1 and 4, structural elements of the actin cytoskeleton with well-characterized roles in cell migration were minimally affected by exposure to FC101 (13,14). It should be stated that although FC101 appeared to have little affect on α-actinin 1 and 4, the disruption of subcellular actin filament structures (leading edge, trailing edge) important for cell migration may underlie the affects of FC101 on glioblastosma cell migration, even in the absence of significant changes in protein expression. Additionally, the antimigratory response of glioblastomas to FC101 may be causally related to the down-regulation of metalloproteinases, as observed in osteosarcomas and mesothelioma cells treated with the fungal metabolites, 3-*O*-methylfunicone and fucoxanthin(20,21). Furthermore, to the best of our knowledge, this is the first study that illustrates the antimigratory effects of FC101 on tumor cells.

In the present study, we demonstrate the effectiveness of FC101 as an anticancer agent in glioblastomas, expanding its potential therapeutic utility for the treatment of human cancers. The prospective application of FC101 for the treatment of cancer is furthered by experimental studies that demonstrate that cancer cells are more sensitive to this fungal metabolite than normal tissue (7,8), suggesting that FC101 circumvents normal tissue toxicity. This therapeutic characteristic of FC101 is particularly advantageous when considering neurotoxicity is often a limiting factor for chemotherapeutic drugs used for the treatment of glioblastomas. Additional caveats to the clinical treatment of these tumors is their therapeutic resistance attributed in part to high recurrence rates that have made combinatorial therapy approaches essential for the management and treatment of this disease. FC101 represents a novel compound that can be explored for its therapeutic applications as a combinatorial agent that augments surgical and radiotherapy approaches for the treatment of glioblastomas.

## Abbreviations

FC101: fusarochromanone
ROS: reactive oxygen species
PARP: poly(ADP-ribose) polymerase
